# Rate of aspiration pneumonia in hospitalized Parkinson’s disease patients: a cross-sectional study

**DOI:** 10.1186/s12883-015-0362-9

**Published:** 2015-07-05

**Authors:** Daniel Martinez-Ramirez, Leonardo Almeida, Juan C. Giugni, Bilal Ahmed, Masa-aki Higuchi, Christopher S. Little, John P. Chapman, Caroline Mignacca, Aparna Wagle Shukla, Christopher W. Hess, Karen Wheeler Hegland, Michael S. Okun

**Affiliations:** Department of Neurology, University of Florida College of Medicine, Center for Movement Disorders and Neurorestoration, Gainesville, FL USA; Department of Neurosurgery, University of Florida College of Medicine, Center for Movement Disorders and Neurorestoration, Gainesville, FL USA; Department of Speech Language and Hearing Sciences, University of Florida College of Public Health and Health Professions, Gainesville, FL USA

**Keywords:** Hospitalization, Pneumonia, Dysphagia, Swallow therapy, Prevention

## Abstract

**Background:**

Aspiration pneumonia is an important cause of morbidity and mortality in Parkinson’s disease (PD). Clinical characteristics of PD patients in addition to specific alterations in swallowing mechanisms contribute to higher swallowing times and impairment in the effective clearance of the airway. These issues may render patients more prone to dysphagia and aspiration events. We aimed to determine the frequency of aspiration events in a hospitalized PD cohort, and to report the number of in-hospital swallow evaluations.

**Methods:**

A retrospective single center chart review of 212 PD patients who had 339 hospital encounters was performed from January 2011 to March 2013. Demographics, clinical characteristics, and reasons for encounters were documented. The number of in-hospital aspiration events and the number of swallowing evaluations and also the implementation of aspiration precautions were recorded.

**Results:**

The cohort had a mean age of 74.1 (SD = 10.1) years with mean disease duration of 6 (SD = 6.3) years. Fifty-two hospital encounters (15.3 %) were related to a pulmonary cause. In-hospital aspiration pneumonia events were reported in 8 (2.4 %) of the total encounters. Swallow evaluations were performed in 25 % of all cases, and aspiration precautions were initiated in 32 % of the encounters. The data revealed that 1/8 patient had swallowing evaluations performed prior to an aspiration event.

**Conclusions:**

In-hospital aspiration pneumonia events were reported in 2.4 % of the hospitalized PD cohort. Preventive measures and precautions were not routinely performed, however rates of aspiration were relatively low. The results highlight the need for more research into screening and monitoring of swallowing problems in PD patients during hospital encounters.

## Background

Parkinson’s disease (PD) is a neurodegenerative disorder characterized by the presence of cardinal motor symptoms such as bradykinesia, tremor, rigidity, and loss of postural reflexes [1]. Over the last few decades, there has been an expanded concept recognizing numerous non-motor characteristics of PD beyond the usual motor symptoms, including neuropsychiatric disorders, sleep disturbances, autonomic, sensory, and gastrointestinal symptoms [2]. These non-motor symptoms become more prevalent as the disease advances and have been cited as a major cause of disability. The recognition of these symptoms will likely be critical for improving healthcare for the expanding PD population [3, 4].

Dysphagia in PD has been shown to be associated with aspiration pneumonia, which is one of the potential reasons for PD hospitalization [5, 6]. Swallowing is a sensorimotor process involving automatic and volitional systems, where decreased cough sensitivity and elevation and excursion of the hyolaryngeal complex have been considered important causes of penetration and aspiration in PD patients [7, 8]. PD patients experience higher rates of hospital-based complications, and often suffer substantial clinical deterioration while hospitalized [9, 10]. It has been estimated that PD patients have a 3.8 times increased risk of developing aspiration pneumonia when compared to the general population [11]. Aspiration pneumonia has been reported as the most frequent cause of death in PD patients, and it has been estimated to account for 70 % of the mortality [12]. The recognition of potentially preventable risk factors and interventions to reduce morbidity, mortality, and economic burdens are important and relevant to this population. We aimed to evaluate the frequency of aspiration events in patients with PD during hospital encounters, and we documented the care practices for these patients.

## Methods

The University of Florida (UF) Institutional Review Board (IRB) reviewed and approved the study. Patient records were de-identified prior to analysis.

### Study design, settings, and population

A dataset of 339 hospital encounters from 212 patients followed at the UF Center for Movement Disorders and Neurorestoration (CMDNR) was used. The dataset included the period from January 2011 to March 2013 and the study was conducted as a retrospective cross-sectional data/chart review. A diagnosis code (*International Classification of Diseases, Ninth Revision, Clinical Modification*) was used to identify subjects with PD (*ICD-9-CM* code 332.0) who had a hospital encounter and this was used as criteria for inclusion into the study. A hospital encounter was defined as an emergency room visit with subsequent hospitalization, or any direct admission to the hospital due to any cause with the exception of admission for elective procedures.

### Data collection

An electronic health record system (EPIC) was used to obtain the basic demographics and clinical data elements from each subject’s chart at the time of the hospital encounter, including results from swallow evaluations and if aspiration precautions were administered. Additionally, the reason for hospital encounter, length of hospital stay in days, and whether a neurologist was consulted during these encounters were also documented. LED was calculated using the following formula [13]: regular LD dose + LD CR dose × 0.75 + LD × 0.33 if entacapone + pramipexole dose × 100 + ropinirole dose × 20 + rotigotine dose × 30 + pergolide dose × 1 + bromocriptine dose × 10 + selegiline dose × 10 + rasagiline dose × 100 + amantadine dose × 1. The Charlson index score, which has previously been used in studies with PD patients [14] was chosen to evaluate the magnitude of impact of each of the comorbidities on the mortality risk [15]. The Charlson index predicts the ten-year mortality for each patient based on comorbidities. Each condition is assigned a score of 1, 2, 3, or 6, depending on the risk of dying associated with each one. Scores are summed to provide a total score to predict mortality. Conditions screened by the Charlson index are cardiovascular disorders, pulmonary diseases, cerebrovascular diseases, connective tissue diseases, liver and kidney diseases, diabetes, dementia, cancer, and/or AIDS. We further divided patients into subgroups including those with or without alteration in mental status according to the physicians clinical examination note at the time of evaluation. Reasons for hospital encounters were categorized based on the Emergency Severity Index (ESI) triage algorithm [16] which classifieds the documented reasons for hospitalization. According to this algorithm, an ESI 1 and 2 were classified as a high risk for a prolonged length of stay, including falls/fractures, pulmonary causes (such as chronic obstructive pulmonary disease, cancer, pulmonary arterial hypertension, fibrosis, vascular disease, pneumonia), cardiac issues/syncope, genitourinary infections, encephalopathy/drug-induced psychosis, cancer, stroke, and dementia, and an ESI 3–5 was classified as low risk, and was inclusive of general medical conditions (including weakness, pain, bleeding, or anorexia), other medical issues (including seizures, vascular problems, or trauma), and gastrointestinal issues.

### Outcome measurements

The primary outcome was the frequency of aspiration pneumonia in a cohort of PD patients during their hospital encounter. Also collected were the circumstances for the aspiration event, including clinical features of the individual and environmental features such as the occurrence of swallow evaluations and aspiration precautions.

To identify subjects who developed aspiration pneumonia during the encounter (in-hospital aspiration) we used a diagnosis code (*ICD-9-CM* code 507.0, 507.8, and 997.32) and performed a manual chart review of each subject and each hospital encounter in order to identify aspiration pneumonia events that occurred at 48 h or greater after the hospital encounter. The frequency of aspiration pneumonia was calculated by dividing the number of hospital-acquired aspiration pneumonias by the total number of hospital encounters, and this was then multiplied by 100. A brief description of the swallowing evaluations and aspiration precautions is provided in following paragraphs.

### Clinical or bedside swallow evaluation (CSE)

The CSE was performed for all patients referred for a swallow evaluation. The assessment included: 1. Patient awake/oriented/able to follow simple commands, 2. Ability to produce sustained phonation and comment on vocal quality, 3. Ability to produce voluntary cough on command, 4. Ability to produce saliva, swallow on command, and observation of hyolaryngeal elevation, 5. Trials of ice-chips: observation of oral bolus manipulation; chew (if present); timeliness of pharyngeal swallow; hyolaryngeal elevation; cough after swallow; voice quality after swallow, and 6. Trials of thin, nectar-thick, honey-thick liquids as deemed appropriate by the speech-language pathologist (SLP): Anterior labial leakage, timeliness of pharyngeal swallow, hyolaryngeal elevation, cough after swallow, and voice quality after swallow.

### Rehabilitation barium swallow evaluation (RBS)

The decision to perform a RBS was made following a CSE and if the patient exhibited signs or symptoms of an impaired swallowing mechanism. These signs included one or more of the following: weak or absent volitional cough, cough after swallow, voice change after swallow, absent volitional swallow, reduced or delayed hyolaryngeal elevation. The RBS was performed in the fluoroscopy suite of the radiology department. Patients were seated upright in the lateral viewing plane, and multiple boluses of thin, thick, and solid consistencies were presented for evaluation by the SLP.

### Aspiration precautions

The recommendation for aspiration precautions was potentially performed before or possibly after the swallow evaluations (whether CSE or RBS). These were typically administered pre-emptively to patients considered by the nurses or physicians to be at-risk for aspiration, but they could also be administered after a swallowing study. The recommendations ranged from nothing by mouth (NPO) in extreme cases of disordered swallowing, or included modifications such as supervision during eating/drinking, sitting upright in a chair, thickened liquids, crushed pills, IV administered pills/medications, or various “dysphagia diets” (*i.e.*, mechanical soft, puree, etc.).

### Statistical analysis

A Mann–Whitney *U* Test was used to compare demographics and continuous variables (age, Charlson index, disease duration, levodopa equivalent dosage, days from admission to swallow evaluations and aspiration precautions, and length of hospital encounter) between those who developed aspiration pneumonia versus those who did not develop aspiration pneumonia. A Chi square test using an Exact Test was performed to compare categorical variables (gender, H&Y stage, and neurology consultation) between populations and to learn whether preventive swallowing measures had an effect on developing aspiration pneumonia during the hospital encounter. Statistical analyses were performed using commercially available statistical software (SPSS, version 19.0; SPSS, Inc., Chicago, Illinois) with a priori alpha levels of ≤ 0.05.

## Results

### Characteristics of population

Sixty-one percent were male, with a mean age of 74.1 (SD = 10.1) years, and a mean Charlson index of 5.9 (SD = 1.5). The cohort had mean disease duration of 6.4 (SD = 6.3) years and had mean LED of 502.4 (SD = 487.9) mg. A neurologist was consulted in 24.5 % and the mean length of hospital stay was of 6 (SD = 7.1) days. No demographic and clinical significant differences were found when comparing those who developed aspiration pneumonia during hospital encounter versus those who did not develop aspiration pneumonia, as shown in Table 1. A pulmonary reason was the cause leading to a hospital encounter in 52/339 (15.3 %). The reasons for hospital encounters have been described in Table 2.

**Table 1:** Demographic and clinical comparison between populations

	Total (n = 339)	Aspiration pneumonia (n = 8)	Non-aspiration pneumonia (n = 331)	P
Age, y (SD)	74.1 (10.1)	76.6 (12.4)	74.1 (10.1)	0.62^a^
Male, no. (%)	206 (60.8 %)	4 (50)	202 (61)	0.72^b^
Charlson index, no. (SD)	5.9 (1.5)	5.9 (1.1)	5.9 (1.5)	0.90^a^
Disease duration, y (SD)	6.4 (6.3)	3.8 (2.2)	6.4 (6.3)	0.59^a^
Hoehn and Yahr stage IV-V, no. (%)	165 (48.7)	4 (50)	161 (48.6)	1.00^b^
LED, no. (SD)	502.4 (487.9)	716.6 (488.5)	496.8 (487.5)	0.15^a^
Neurologist consulted, no. (%)	83 (24.5)	1 (12.5)	82 (24.8)	0.71^b^
Length of hospital stay, days (SD)	6 (7.1)	4.3 (3.6)	6.0 (7.2)	0.56^a^
Swallow evaluations or aspiration precautions, no. (%)	158 (46.6)	7 (87.5)	1 (12.5)	0.03^b^
Days from admission to barium evaluation, no. (SD)	4.5 (4.3)	4.3 (4.3)	4.5 (4.4)	0.98^a^
Days from admission to clinical evaluation, no. (SD)	3.5 (4.7)	4.3 (2.2)	3.5 (4.8)	0.13^a^

^a^Mann–Whitney *U* Test

^b^Fisher’s Exact Test

**Table 2:** Reasons for hospital encounters (n = 339)

	Frequency	Percentage
Falls/fractures	61	18
Pulmonary	52	15.3
General medical problems	51	15
Other	48	14.2
Gastrointestinal issues	43	12.7
Encephalopathy/drug-induced psychosis	24	7.1
Cardiac issues/syncope	22	6.5
Stroke	19	5.6
Genitourinary infections	13	3.8
Cancer	6	1.8

### Aspiration pneumonia, swallow evaluations and aspiration precautions

Aspiration pneumonia events were reported in 8 (2.4 %) hospital encounters of 8 individual patients. The reasons for hospital encounters were: 3 due to general medical problems, 2 due falls/fractures, 2 due to other pulmonary causes, and 1 due to other reasons. Seven subjects of the 8 who developed aspiration pneumonia during the hospital encounter were considered as having a high-risk reason of admission, *X*^2^ (2) = 2.9, p = 0.09. Two of the 8 subjects had altered mental status at admission and there was no significant difference in the frequency of aspiration events when compared to those without alteration, *X*^2^ (2) = 0.36, p = 0.72. Additionally, 6/52 (11.5 %) hospital encounters that were related to pulmonary causes were due to an aspiration pneumonia acquired outside the hospital.

The swallow evaluations were performed in 83/339 (24.5 %) while aspiration precautions were adopted in 116/339 (34.2 %) of the hospital encounters. The mean days from admission until swallow evaluation was of 4.5 (SD = 4.3) for RBS and 3.5 (SD = 4.7) for CSE. A greater percentage of swallow evaluations and aspiration precautions was performed on those who developed in-hospital aspiration pneumonia, 7/8 (87.5 %) vs. 1/8 (12.5 %), *X*^2^ (2) = 5.51, p < 0.03. The evaluations and/or precautions were implemented after the occurrence of aspiration pneumonia in 7/8 cases. Two of the 8 in-hospital aspiration pneumonia events developed after an intubation procedure.

## Discussion

This retrospective cross-sectional review of 339 hospital encounters revealed a frequency of in-hospital aspiration pneumonia of 2.4 %. Studies have reported an estimated incidence of community-acquired pneumonia in PD patients varying between 11–60 % [17, 18], but there has been a lack of data on hospitalized patients.

This cohort demonstrated a surprisingly low frequency of in-hospital swallowing evaluations and also there was a low amount of education offer in hospital for aspiration precautions. Swallowing evaluations were ordered in less than 25 % of cases, and there was a mean delay from admission of 3–5 days. Aspiration precautions were administered in less than 35 % of the hospital encounters. Swallow evaluations were more frequently implemented following the discovery of hospital-acquired aspiration pneumonia. A closer inspection of the data revealed that two of the eight in-hospital aspiration pneumonia events developed after an intubation procedure.

The original reports of swallowing complications in PD were published in the 1980s [19], and since that time there has been a recent interest in aspiration in this population, and this interest has been magnified by the published associations of aspiration with morbidity and mortality. PD patients without symptoms of dysphagia have prolonged swallowing times [20, 21] and impairments in the ability to effectively clear the airways [7, 22, 23]. These attributes render these patients more prone to developing dysphagia and to subsequent aspiration. Recent studies have also detected microscopic neural degeneration, regenerative changes [24], and deposition of alpha-synuclein in the pharyngeal structures [25], and these could possibly be the pathophysiological underpinnings of swallowing abnormalities in PD.

Interestingly, the demographic, clinical characteristics, or mental status at admission correlated with aspiration events. However, we did observe a tendency for developing aspiration pneumonia in high-risk patients with prolonged length of stay. In the future it will be important to detect high-risk PD patients for aspiration at the time of the hospital admission. Awareness among hospital staff and physicians and the implementation of preventative measures could be a critical change in practice. Whether addressing these issues could decrease the incidence of aspiration remains unknown.

Preventative measures have been proposed as fundamental for decreasing aspiration-related morbidity and mortality in PD [26, 27]. Swallowing function usually does not respond to levodopa therapy, however in some cases it may be partially improved [28], non-pharmacological measures [29] such as providing adequate nutrition without sacrificing airway safety are also important. There are no standardized guidelines for the management of the hospitalized PD patients and better management protocols are needed [30, 31].

There were several limitations of this study. The retrospective nature of the data obtained from ER clinical notes could have possibly resulted in missing aspiration cases and inaccurate staging of the disease. Additionally, the cognitive profile was not well documented for all patients. Second, we based the diagnosis of aspiration pneumonia on the physician’s clinical note. The use of microbiological cultures from pulmonary aspirates could have possibly improved diagnostic certainty. Third, the data was culled from a referral center of movement disorders and all patients were followed in an expert PD clinic, which routinely performs swallow evaluations, and this may have biased the low rate of aspiration events. Finally, the use of non-validated stratifying indexes (in PD populations) may have limited the validity of the data. The assessment of length of hospital stay was based on the Charlson index, which consider various comorbidities. We were not able to control for these comorbidities including dementia [32] and to control for stroke or diabetes [33]. These comorbidities can impact risk of aspiration. The comorbidity issue could not be completely addressed due to a lack of statistical power for such an analysis. Finally, the population had a mean disease duration of 6 years, which could have possibly underestimated the incidence, and magnitude of the results.

## Conclusions

In summary, though in-hospital aspiration pneumonia events were reported to be low in our PD cohort this should not diminish the importance of these events. Swallowing evaluations and precautions were routinely underutilized. Additional larger prospective studies from a community hospital sample will be necessary to better understand risk factors, preventative strategies, and also longitudinal follow-up of in-hospital aspiration events.
